# Frequency of Rare Allelic Variation in Candidate Genes among Individuals with Low and High Urinary Calcium Excretion

**DOI:** 10.1371/journal.pone.0071885

**Published:** 2013-08-26

**Authors:** Hakan R. Toka, Giulio Genovese, David B. Mount, Martin R. Pollak, Gary C. Curhan

**Affiliations:** 1 Division of Nephrology, Brigham and Women's Hospital, Boston, Massachusetts, United States of America; 2 Division of Nephrology, Beth Israel Deaconess Medical Center, Harvard Medical School, Boston, Massachusetts, United States of America; 3 Channing Division of Network Medicine, Boston, Massachusetts, United States of America; University of Texas School of Public Health, United States of America

## Abstract

Our study investigated the association of rare allelic variants with extremes of 24-hour urinary calcium excretion because higher urinary calcium excretion is a dominant risk factor for calcium-based kidney stone formation. We resequenced 40 candidate genes potentially related to urinary calcium excretion in individuals from the Nurses' Health Studies I & II and the Health Professionals Follow-up Study. A total of 960 participants were selected based on availability of 24-hour urine collection data and level of urinary calcium excretion (low vs. high). We utilized DNA sample pooling, droplet-based target gene enrichment, multiplexing, and high-throughput sequencing. Approximately 64% of samples (n = 615) showed both successful target enrichment and sequencing data with >20-fold deep coverage. A total of 259 novel allelic variants were identified. None of the rare gene variants (allele frequencies <2%) were found with increased frequency in the low vs. high urinary calcium groups; most of these variants were only observed in single individuals. Unadjusted analysis of variants with allele frequencies ≥2% suggested an association of the Claudin14 SNP rs113831133 with lower urinary calcium excretion (6/520 versus 29/710 haplotypes, P value = 0.003). Our data, together with previous human and animal studies, suggest a possible role for Claudin14 in urinary calcium excretion. Genetic validation studies in larger sample sets will be necessary to confirm our findings for rs113831133. In the tested set of candidate genes, rare allelic variants do not appear to contribute significantly to differences in urinary calcium excretion between individuals.

## Introduction

Kidney stone disease is a major cause of morbidity associated with tremendous pain, suffering, and substantial economic impact [1]–[3]. The majority of kidney stones contain calcium, most commonly in the form of calcium (Ca^2+^) oxalate. Higher urinary Ca^2+^ excretion is associated with higher risk of calcium-containing kidney stone formation. The etiology of the vast majority of cases of hypercalciuria is unknown and is referred to as idiopathic hypercalciuria [4].

Nephrolithiasis is a multifactorial disease with genetic and environmental factors determining the likelihood of stone formation [5]. We and other investigators have identified multiple environmental risk factors associated with increased risk including lower dietary Ca^2+^ intake [6]–[8], lower fluid intake [6]–[9], and higher body mass index [8], [10]. A family history of nephrolithiasis is associated with a greater than two-fold increase in the risk of developing a stone [11]. Substantial data demonstrate that calcium-based kidney stones and elevated urinary Ca^2+^ are linked and likely have a strong genetic component [12]. Genes for rare Mendelian forms of nephrolithiasis and increased urinary Ca^2+^ excretion have been identified; however, mutations in these genes explain only a very small fraction of kidney stone disease in the general population. For example, mutations in the renal chloride channel CLCN5 cause Dent's disease, a group of X-linked hypercalciuric abnormalities [13]. Inactivating (loss-of-function) mutations in the calcium-sensing receptor (CaSR) cause autosomal-dominant hypocalcemia and hypercalciuria [14]. Another example is familial hypomagnesemia associated with hypercalciuria and nephrocalcinosis (mutations in Claudin16) [15]. Additional genes were identified in various animal models such as the renal epithelial Ca^2+^ transporter gene (TRPV5), which when mutated causes severe hypercalciuria in the mouse [16].

Rather than testing the “common disease, common variant” hypothesis pursued by genome wide association studies (GWAS), our approach tested the association of the frequencies of rare genetic variants with 24-hour urinary Ca^2+^ excretion [17]. This approach has succeeded when examining other complex traits such as hypertriglyceridemia [18], hypercholesterolemia [19], and non-alcoholic fatty liver disease [20]. The findings in these studies were consistent with *in silico* predictions that some sequence variations found in healthy individuals are as deleterious to protein function as mutations that, in other genes, cause monogenic disease. Highly penetrant rare alleles may be an important genetic contributor to common disease seen in the general population as shown for blood pressure variation [21].

The goal of this work was to identify rare, functionally significant genetic variants associated with urinary Ca^2+^ excretion. Forty candidate genes possibly related to urinary Ca^2+^ excretion were resequenced at extremes of urinary Ca^2+^ excretion in 960 individuals from three well-characterized cohorts, the Nurses' Health Studies (NHS) I & II and the Health Professional Follow-Up Study (HPFS).

## Methods

### A. Study cohorts

The NHS I was established in 1976 with over 120,000 female registered nurses aged 30–55 years. The NHS II was established in 1989 with over 116,000 female nurses aged 25–42 years. The HPFS was established in 1986 with over 51,000 male health care professionals aged 40–75 years. All three cohorts have been followed by biennially mailed questionnaires including questions on lifestyle practices and newly diagnosed diseases such as nephrolithiasis [22]. Additional information was obtained from self-reported cases including symptoms and kidney stone type. In validation studies, permission to obtain medical records was requested from newly diagnosed cases in all three cohorts. The diagnosis of stone disease was confirmed in over 90% of these cases. Twenty-four-hour urine collections were obtained from participants with a history of confirmed nephrolithiasis and from randomly selected controls. Those with a history of kidney stones performed the collections after the diagnosis. All 24-hour urine collections were performed using the Mission Pharmacal system (San Antonio, TX, USA). Urinary Ca^2+^ was measured by an atomic absorption spectrophotometer [22]. Approximately 10% of individuals with the highest and lowest values of 24-hour urinary Ca^2+^ excretion from available male and female participants in equal numbers were selected.

This study was approved by the Brigham and Women's Hospital's institutional review board (approval # 2000P001316). The institutional review board (IRB) specifically considered the risks and anticipated benefits, if any, to participants, and the selection, safety and privacy of individuals. Implied consent was considered as appropriate by the IRB for this specific study.

### B. DNA samples

DNA samples were collected as part of a general collection of blood samples in the three cohort studies. We limited this study to those who self-reported their race as Caucasian (and this was confirmed as part of a separate GWAS analysis). High quality DNA was extracted (Dana Farber/Harvard Cancer Center) from buffy coats via QIamp 96 spin-protocol (Quiagen Inc., Chatsworth, CA). DNA concentrations were calculated in 96-well format using a Molecular Dynamics spectrophotometer. The 960 DNA samples were ranked by urinary Ca^2+^ excretion and grouped into pools of 15 or 20 samples prior to target DNA capture. A pilot project testing pooled samples of 15 versus 20 individual samples did not show any differences in DNA capture efficiency and sequence coverage per individual samples between the pools (data not shown). We subjected 52 sample pools (N = 960 individuals; 16 pools of 15 samples and 36 pools of 20 samples) to RainDance target (RDT) DNA capture (**Table 1**). The amount of genomic DNA provided for RDT capture per pool was 10 µg (0.5–0.66 µg per individual sample).

**Table 1:** DNA pooling strategy for 960 individuals with low (n = 480) vs. high (n = 480) urinary Ca^2+^ excretion.

Cohort	No. of pools (sets of 15+20 samples)	No. of individuals
NHS I, low urinary Ca^2+^	7 (4×15+3×20)	120
NHS II, low urinary Ca^2+^	6 (6×20)	120
HPFS, low urinary Ca^2+^	13 (4×15+9×20)	240
NHS I, high urinary Ca^2+^	7 (4×15+3×20)	120
NHS II, high urinary Ca^2+^	6 (6×20)	120
HPFS, high urinary Ca^2+^	13 (4×15+9×20)	240

NHS = Nurses' Health Study; HPFS = Health Professional Follow-up Study.

### C. Candidate genes

Candidate genes were selected by searching public databases (PubMed and Online Mendelian Inheritance in Man (OMIM)). We limited the number of genes to 40 due to cost and technical limitations at the time of study design. Our priority was to achieve sufficient sequence coverage (at least 20×) to detect rare allelic variation. Selection of candidate genes was based on *in vivo* and *in vitro* evidence of regulating Ca^2+^ homeostasis in bone, kidney or intestine. We also selected some genes, such as oxalate and citrate exchangers, which may affect calcium-based stone formation by changing supersaturation rather than urinary Ca^2+^ excretion per se. The genes resequenced in this study are listed in **Table 2**, including gene name, gene/protein function, RefSeq ID and exon number. References underlining the rationale of gene selection are provided. As an additional gene, we included *PIK3C2G* (phosphoinositide-3-kinase, class 3, gamma polypeptide) based on our unpublished GWAS data. PIK3C2G regulates diverse cellular responses, such as cell proliferation, oncogenic transformation, cell migration, intracellular protein trafficking, and cell survival.

**Table 2:** Candidate genes included in the resequencing study (n = 40).

Gene	Gene name/protein function	RefSeq ID	Exons
Aconitase [32]	Catalyzes the isomerization of citrate to isocitrate	NM_001098	18
CaSR [33]	Calcium-sensing receptor	NM_000388	7
Citrate lyase [34]	Catalyzes the formation of acetyl-CoA from citrate	NM_001096	29
Claudin 2 [35]	Tight junction protein, proximal tubule (PT)	NM_001171092	2
Claudin 8 [35]	Tight junction protein, primarily distal nephron (DCT)	NM_99328	1
Claudin 10 [36]	Tight junction protein, thick ascending limb (TAL) and intestine	NM_001160100	5
Claudin 14 [37]	Tight junction protein, TAL	NM_001146077	3
Claudin 16 [15]	Tight junction protein, TAL	NM_006580	4
Claudin 19 [30]	Tight junction protein, TAL and DCT	NM_001123395	5
CLCN5 [38]	Chloride channel 5, mutations cause Dent's disease	NM_000084	15
CLCNKA [39]	Basolateral chloride channel expressed in TAL	NM_001042704	20
CLCNKB [40]	Basolateral chloride channel expressed in TAL and DCT; mutation cause type III Bartter's syndrome	NM_000085	20
FGF23 [41]	Fibroblast growth factor 23, phosphatonin	NM_020638	3
GCMB [42]	Glial cell missing B, mutations cause familial isolated hypoparathyroidism	NM_004752	5
Klotho [43]	Regulator of TRPV5 and FGF23	NM_004795	5
NHERF1 [44]	Hydrogen exchanger regulatory factor 1, mutations cause hypophosphatemia and nephrolithiasis	NM_004252	6
NHERF2 [45]	Hydrogen exchanger regulatory factor 2, expressed like NHERF1 in PT	NM_001130012	7
NKCC2 [46]	Na^+^-K^−^-2Cl^−^-cotransporter, mutations cause type 1 (neonatal) Bartter's syndrome	NM_000220	27
PDZK1 [47]	Hydrogen exchanger regulatory factor 3, PT	NM_002614	10
PIK3C2G	Phosphoinositide-3-kinase, class 3, γ polypeptide	NM_004570	32
PTH [48]	Parathyroid hormone	NM_000315	3
PTHR [49]	Parathyroid hormone receptor 1	NM_000316	16
ROMK [50]	Renal outer medullary K^+^ channel, mutations cause type 2 Bartter's syndrome	NM_000338	4
SLC12A3 [51]	Thiazide-sensitive Na^+^-Cl^−^ cotransporter, mutation cause Gitelman's syndrome	NM_000339	26
SLC13A2 [52]	Na^+^ citrate transporter NaC1	NM_001145975	12
SLC13A3 [53]	Na^+^ citrate transporter NaC2	NM_001193340	14
SLC25A1 [54]	Mitochondrial citrate transporter	NM_005984	9
SLC26A1 [55]	Oxalate and sulfate anion transporter	NM34425	4
SLC26A2 [56]	Oxalate and citrate exchanger	NM_000112	2
SLC26A6 [57]	Oxalate and citrate exchanger	NM_001040454	21
SLC34A1 [58]	Na^+^ phosphate co-transporter NaPi2A	NM_003052	13
SLC34A3 [59]	Na^+^ phosphate co-transporter NaPi2C	NM_001177316	13
SLC4A1 [60]	AE1 oxalate, mutations cause distal RTA	NM_000342	20
SLC4A2 [61]	AE2 oxalate	NM_003040	23
SLC4A3 [61]	AE3 oxalate	NM_005070	23
TRPV5 [62]	Epithelial Ca^2+^ channel ECaC1	NM_019841	14
TRPV6 [63]	Epithelial Ca^2+^ channel ECaC2	NM_018646	15
UMOD [64]	Uromodulin	NM_001008389	11
VDR [65]	Vitamin D receptor	NM_000376	11
WNK4 [66]	Protein kinase, lysine deficient 4, mutations cause pseudohypoaldosteronism type 2	NM_032387	19

### D. Primer design

Our list of the 40 genes was provided to RainDance Technologies (RDT) (Lexington, MA) for custom primer design based on the Primer3 algorithm (http://frodo.wi.mit.edu/primer3). The custom panel was prepared and primers were designed to target all 497 exons of the 40 candidate genes, including ∼50 bp of intronic sequence flanking each exon. The 795 amplicons in the panel ranged in size from 200 to 600 bases, with a GC content of 25% to 87%, and represented a total coding sequence of ∼182 kb. All single nucleotide polymorphisms (SNPs) and repeat regions were filtered from the primer selection region. The RDT design was quality checked to ensure that none of the primers were designed over known SNPs and primer sequences were verified to avoid repetitive regions of the genome using the program RepeatMasker (http://www.repeatmasker.org). The primers for the 795 amplicons varied in annealing temperature from 57°C to 60°C, with a primer length range of 15 to 22 bases. Other rules for primer design included BLASTing the primers to the chromosome that contained the gene of interest and *in silico* PCR to match the designed primers to PCR product and target sequence.

### E. Enrichment of target DNA for sequencing

The capture was performed at two laboratories, RDT in Lexington, MA, and Ambry Genetics in Aliso Viejo, CA. DNA samples were fragmented to 3 to 4 kb by shearing the genomic DNA with the Covaris S2 instrument (Covaris, Woburn, MA) following the manufacturer's instructions. To prepare the input DNA template mixture for targeted amplification, 3 µg of the purified genomic DNA fragments were added to 4.7 µL of high-fidelity buffer (Invitrogen, Carlsbad, CA), 1.26 µL of magnesium sulfate (Invitrogen), 1.6 µL of 10 mmol/L dNTP (Invitrogen), 3.6 µL of 4 mol/L betaine (Sigma-Aldrich, St. Louis, MO), 3.6 µL of Droplet Stabilizer (RDT, Lexington, MA), 1.8 µL of dimethyl sulfoxide (Sigma-Aldrich), and 0.7 µL of 5 units/µL of Platinum High-Fidelity Taq (Invitrogen). The samples were brought to a final volume of 25 µL with nuclease-free water. PCR droplets were generated on the RDT1000 instrument. The enrichment panel consisted of an emulsion that contained a collection of unique primer droplets in which each primer droplet contained a single matched forward and reverse primer for each amplicon in the panel. Each panel contained multiple replicates of each unique primer droplet with consistent volume. The RDT1000 generated a PCR droplet by pairing a single genomic DNA template droplet with a single primer droplet. The paired droplets flowed past an electrode in the RDT chip and were instantly merged to create a single PCR droplet. All of the resulting PCR droplets were dispensed as an emulsion into a PCR tube and then transferred to a standard thermal PCR cycler for amplification (Gene-Amp 9700 thermocycler, Applied Biosystems, Foster City, CA). After PCR amplification, the emulsion was broken to release each individual amplicon from the PCR droplets. For each sample, an equal volume of Droplet Destabilizer (RDT) was added to the emulsion of PCR droplets, the sample was vortexed for 15 sec, and spun in a microcentrifuge at 13,000× g for 5 min. The oil below the aqueous phase was carefully removed from the sample and the remaining sample was purified using a MinElute column (Qiagen, Valencia, CA) following the manufacturer's recommended protocol. The purified amplicon DNA was tested on an Agilent Bioanalyzer (Agilent Technologies, Santa Clara, CA) to confirm that it matches the expected amplicon profile (mixture of amplicons ranging from 200 to 600 bp in size).

### F. Targeted deep DNA sequencing

**Figure 1:**
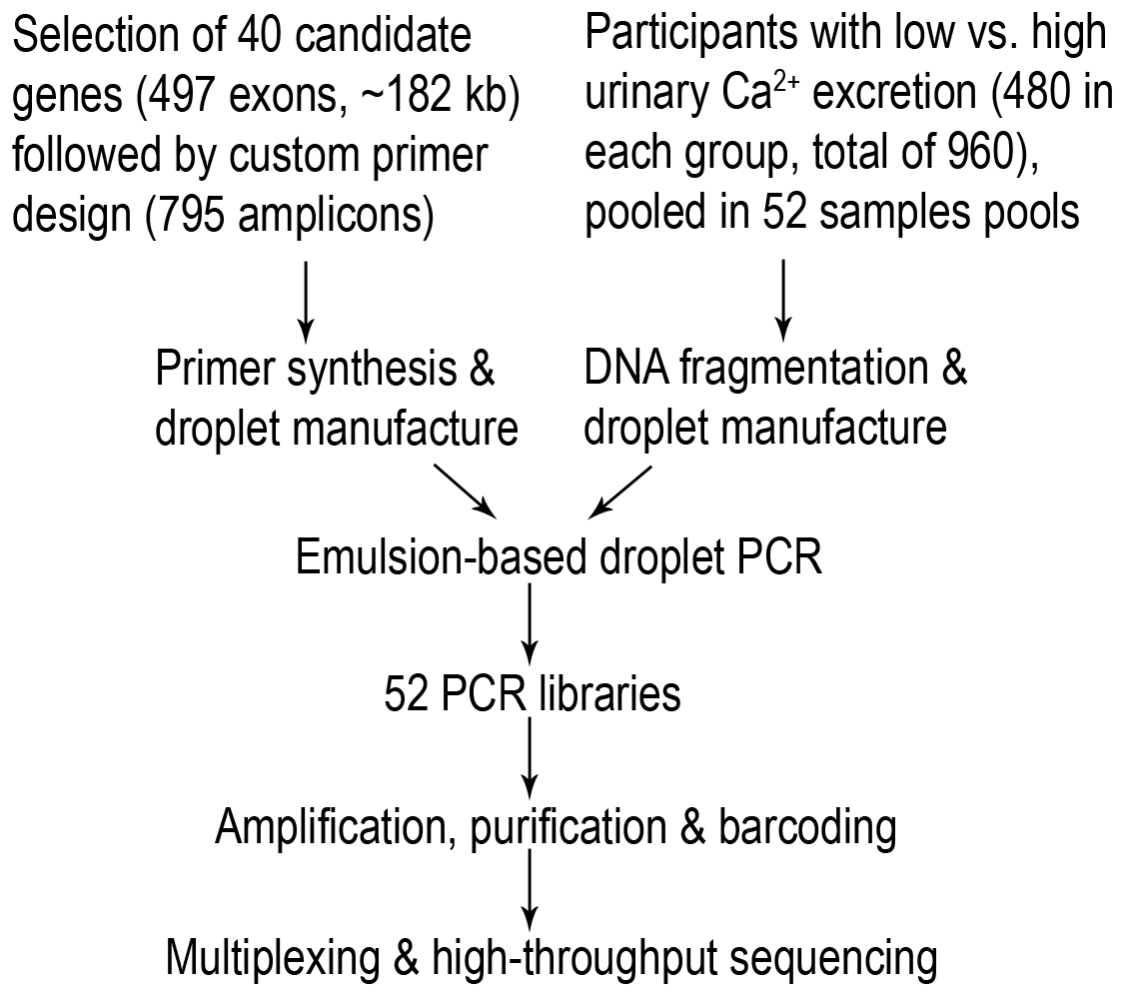
Simplified schematic illustration of the study design utilizing emulsion-based droplet PCR technology and next-generation sequencing.

A simplified schematic illustration of our emulsion-based droplet PCR approach and the off-chip work flow before high-throughput sequencing is presented in **Figure 1**. Successfully enriched sample pools were barcoded and sequenced on the Hiseq2000 Illumina platform (7 barcoded sample pools per lane). After PCR purification, amplified fragments for each individual were repaired to blunt ends using NEB Quick blunting kit (NEB, catalog # E1201L, 15 min RT). The PCR fragments were then linked using NEB Quick ligation kit (NEB, catalog # M2200L). Ligation was done overnight at 25°C. The ligated products were made into 100 µL volume by adding elution buffer and were then sheared using Covaris E210 (Covaris, Woburn, MA). The sheared fragments were purified using Qiagen QIAquick PCR purification column and eluted in 32 µL of elution buffer. The samples then entered the standard Illumina Genome Analyzer multiplex library introduced preparation protocol. The enrichment was confirmed by running an Agilent BioAnalyzer 7500 DNA chip. A quantitative PCR was done to quantitate the library using KAPA Library quantification kit (KAPA Biosystems, Woburn, MA, USA, catalog # KK4824). Enriched DNA was denatured and diluted to a concentration of 8 pM. Seventy bp single end sequencing was performed using standard IGAII manuals and version 4 kits. Seven sample pools per lane of Illumina sequencing were multiplexed. After sequencing, the reads consisting of 795 fragments covering 262,545 bases were mapped and variants sites identified against the reference sequence using the BWA software [23]. This region is larger than the actual targeted bases (182,345) as RDT included some intronic and intergenic regions to facilitate primer picking. All sequence data aligned for the analysis had sequence coverage exceeding 20-fold. Sequences were compared to those reported by HapMap using a custom perl script to assess the rates of data completion and accuracy. The HapMap data was assumed to be without error when estimating data accuracy.

### G. Variant identification

Utilizing the Syzygy software [24], we compared all identified single nucleotide variants (SNVs) with three publically available SNP databases including dbSNP (http://www.ncbi.nlm.nih.gov/projects/SNP/), 1000 Genome Project (1000G) [25], and Exome Sequencing Project (ESP) (http://evs.gs.washington.edu/EVS/) databases. After filtering known SNPs, we used the transition to transversion ratio (Ti/Tv) to filter out false positive novel variants, which occur frequently on high-throughput sequencing platforms. Ti/Tv can be used to estimate true-positive (TP) to false-positive (FP) SNP data [26]. A transition is the mutation of a purine nucleotide to another purine (A<->G) or a pyrimidine to another pyrimidine (C<->T). In contrast, a transversion (Tv) substitutes a purine for a pyrimidine or vice versa. The initially observed Ti/Tv ratio in our SNV data was a mixture of true-positive and false-positive SNVs. This was based on the assumption that if all detected SNVs are true, Ti/Tv equals ∼3.3. If all SNVs are false Ti/Tv equals 0.5. We assumed that common novel variants were most likely artifacts of sequencing. We therefore considered novel variants only if they were observed less than or equal to 3 times in the whole dataset. In nature, Tis are more common than Tvs. We used the following equation to estimate the number of true positive SNVs: %TP = (Ti/Tvobs-Ti/TvFP)/(Ti/TvTP-Ti/TvFP). Considering that Ti/TvTP is around 2.8–3.3, the %TP should be ∼9–11%.

## Results

In order to query the potential relevance of rare allelic variation in genes associated with urinary Ca^2+^ excretion, we decided to amplify and sequence the exons of 40 genes in 960 well-characterized individuals from the NHS and HPFS populations as outlined in the methods section (see **Tables 1** **and** **2**, **Figure 1**)

### Efficacy of DNA enrichment and sequencing

Forty-two out of 52 samples pools were successfully captured (N = 730 individual samples). Ten sample pools (N = 230) failed enrichment due to technical issues. The 42 samples pools were barcoded and further pooled (in sets of 7 sample pools) for sequencing on the HiSeq2000 Illumina platform. Eight samples pools had insufficient sequence coverage (<2-fold). Thirty four sample pools (N = 615) showed sequencing data with >20-fold sequence coverage. These raw sequence data were deposited into the NIH short read archive (SRA) database (http://www.ncbi.nlm.nih.gov/sra) under the accession number PRJNA209216. The target DNA showed uniform amplification across all target amplicons.

The distribution of study participants with >20-fold sequence coverage from the three cohorts in low (N = 355) and high (n = 260) urinary calcium excretion groups are shown in **Table 3**. The phenotype data provided include urinary solute excretion, age and body mass index (BMI). The number of kidney stone formers was significantly higher in the high urinary Ca^2+^ group (n = 164 vs. n = 96; Chi Square P value = 0.004).

**Table 3:** Study participants with successful DNA target amplification and sequencing.

	Lower urinary Ca^2+^ excretion n = 355 (range 18–165 mg/day)		Higher urinary Ca^2+^ excretion n = 260 (range 210–465 mg/day)	
	Stone formers n = 182	Non-stone formers n = 173	Stone formers n = 164*	Non-stone formers n = 96
Ca^2+^ (mg/day)	100±37	101±35	320±57	309±56
Age (years)	64±11	61±8	59±10	60±8
BMI (kg/m^2^)	28±6	26±5	28±5	27±5

The frequency of kidney stone formers was significantly higher (*) in the high urinary Ca^2+^ group (Chi Square P value = 0.004).

### Allelic variation in targeted DNA sequence

**Figure 2:**
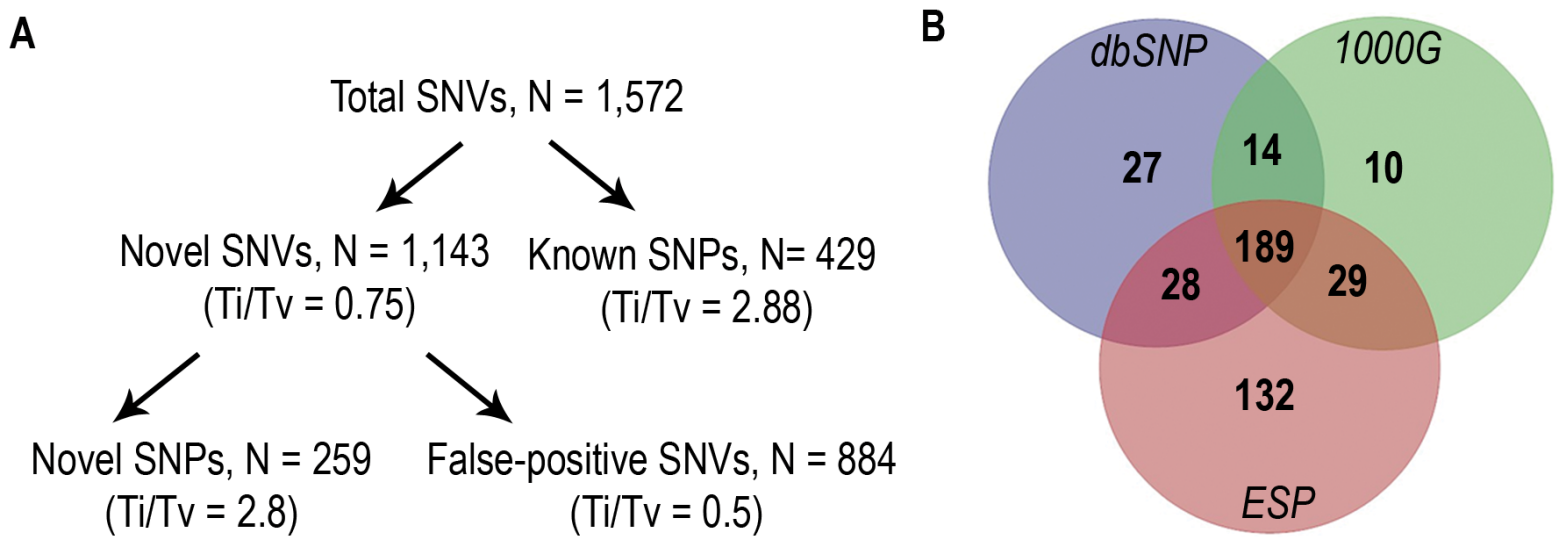
The flowchart data analysis of identified sequence nucleotide variants (SNVs) (**A**). After identifying known SNPs utilizing publically available databases (SNP distribution shown in **B**), false-positive SNVs were filtered based on frequency in our dataset (present four or more times). The transition to transversion ratio (Ti/Tv) of naturally occurring SNPs supported this analytical approach. dbSNP = Single Nucleotide Polymorphism Database; 1000G = 1000 Genome Project; ESP = Exome Sequencing Project.

Samples pools that passed our quality matrix, by enriching for target DNA and showing over 20-fold sequence coverage, were included in our analysis (N = 615 individuals). The total number of identified sequence nucleotide variants (SNVs) with Szygy software was 1,572 (**Figure 2A**). Of these, 429 were known SNPs based on comparison to the three widely used databases (dbSNP, 1000G and ESP databases) (**Figure 2B**). The Ti/Tv of these known SNPs (2.88) differed from the Ti/Tv of the 1,572 SNVs discovered in our study population (0.75). This finding suggested a significant portion of false-positive SNVs. The number of novel variants was reduced to 259 after exclusion of novel SNVs, which were seen 4 or more times and were not present in the tested databases (N = 884, Ti/Tv 0.5). The Ti/Tv of novel allelic variants improved from 0.75 to 2.8, which is both within the expected range of Ti/Tv in naturally occurring mutations (∼2.8–3.3) and similar to the Ti/Tv of the 429 known SNPs (2.88) identified in this study. **Table S1** lists all identified novel SNPs (n = 259) including information on chromosomal location, nucleotide substitution, and effect on protein sequence. Novel silent, nonsense and missense SNPs with existing RefSeq accession numbers were deposited into the NCBI ClinVar database (https://www.ncbi.nlm.nih.gov/clinvar/). ClinVar accession IDs are provided in **Table S1**.

### SNP data for Claudin14

**Table 4** lists all CLDN14 variations identified in our sample set. SNP rs113831133 was more common among individuals in the lower (29 out of 710 haplotypes; 4.1%) compared with the higher (6 out of 520 haplotypes; 1.1%) urinary Ca^2+^ excretion group (Fisher's exact test: P value = 0.003). When adjusted for multiple comparisons with Bonferroni's correction (for n = 429 known SNPs), our finding for rs113831133 did not reach statistical significance (required P value <0.0001). SNP rs11381133 did not vary by sex (17/650 in females, 2.6%, vs. 18/580 in males, 3.1%, P value = 0.61). The common, synonymous CLDN14 SNP rs219780, previously shown to be associated with kidney stone disease in a large GWAS study [27], did not differ between the two urinary Ca^2+^ groups (unadjusted P value = 0.82).

**Table 4:** Distribution of all identified Claudin14 SNPs in the low and high urinary Ca^2+^ excretion groups with unadjusted Chi square P values.

Chr. Position	SNP ID (rs…)	SNP class	Nucleotide	Low urine Ca^2+^	High urine Ca^2+^	P
37833330	.	missense	c.664G>T	2.2% (16/710)	1.5% (8/520)	0.41
37833331	.	silent	c.663G>A	0.0% (0/710)	0.2% (1/520)	-
37833934	.	silent	c.60C>T	0.1% (1/710)	0.2% (1/520)	-
37833931	117560775	silent	c.63G>A	1.4% (10/710)	3.2% (17/520)	0.03
37833694	113350364	silent	c.300C>T	0.4% (3/710)	0.0% (0/520)	-
37833699	.	missense	c.295G>A	0.0% (0/710)	0.3% (1/520)	-
37833751	219779	silent	c.243C>T	24.2% (172/710)	23.8% (124/520)	0.89
37833979	.	silent	c.15C>T	0.0% (0/710)	0.2% (1/520)	-
37833809	.	missense	c.185A>G	0.4% (3/710)	0.0% (0/520)	-
37833892	.	silent	c.102G>A	0.1% (1/710)	0.0% (0/520)	-
37833865	.	silent	c.129C>T	0.3% (2/710)	0.0% (0/520)	-
37833506	.	missense	c.488C>T	0.3% (2/710)	0.2% (1/520)	-
**37833983**	**113831133**	**missense**	**c.11C>T**	**4.1% (29/710)**	**1.1% (6/520)**	**0.003**
37833976	.	silent	c.18G>A	0.3% (2/710)	0.0% (0/520)	-
37833661	74934405	silent	c.333A>C	4.1% (29/710)	4.4% (23/520)	0.77
37833304	.	silent	c.690C>T	0.1% (1/710)	0.1% (1/520)	-
37833307	219780	silent	c.687G>A	18.4% (131/710)	18.9% (98/520)	0.82

The missense variant rs113831133, highlighted in bold, was more frequent among individuals with lower urinary Ca^2+^ excretion. This association did not reach statistical significance when adjusted for multiple comparisons.

## Discussion

The goal of this work was to quantify the frequency of rare, presumably functional genetic variants in individuals with lower and higher urinary Ca^2+^ excretion because urinary Ca^2+^ is a major risk factor for calcium-based kidney stone disease [4]. A different approach has been pursued previously in a large GWAS for kidney stone disease and has shown only limited success possibly due to the complex mechanistic nature of nephrolithiasis. That GWAS in 3,773 kidney stone cases and 42,510 controls from Iceland and the Netherlands reported an association of CLDN14 with nephrolithiasis. The synonymous CLDN14 variant rs219780(C) (minor allele frequency ∼20%) showed a significant association with kidney stone disease (P value = 4×10^−12^) and low bone mineral density (for the hip P value = 0.00039) [27].

In this study, we tested the association of the frequencies of rare allelic variants with possible effect on urinary Ca^2+^ excretion by resequencing 40 known genes that could potentially affect or correlate with urinary Ca^2+^ excretion either in human disease or animal models. Our main hypothesis was that differing number of rare variants between individuals with lower and higher urinary Ca^2+^ excretion would be identified in at least some of these candidate genes. Rare, non-synonymous variants in low and high urinary Ca^2+^ excretion groups could contribute to the level of urinary Ca^2+^ excretion (lowering or increasing excretion and thereby protecting from or predisposing to calcium-based stone formation). This approach has succeeded for other phenotypes such as hyperlipidemia [18],[19] and non-alcoholic fatty liver disease [20], where the frequency of rare gene variants was significantly different in the extremes of the studied phenotype. The results in those studies were consistent with *in silico* predictions that rare amino acid changing sequence variations found in healthy individuals are as deleterious to protein function as gene mutations causing Mendelian disease. Such sequence variations may explain a significant fraction of phenotypic variation in human as suggested for blood pressure variation in the Framingham Heart Study population [21].

We identified 1,572 single nucleotide variants (SNVs), most of which appeared common in our subjects. Initially, we filtered out known gene variants based on three large SNP databases and then applied a simple but very efficient method the Ti/Tv of naturally occurring mutations [26]. We filtered potentially false-positive SNVs, significantly reducing the initially seen number of SNVs and improving the Ti/Tv from 0.75 to a “normal” range of 2.88. Of the 259 novel SNPs in 40 different genes, none showed a significantly increased frequency in the low versus the high urinary Ca^2+^ excretion groups. These extremely rare candidate gene variants identified mostly in single individuals could be functionally contributing to the level of urinary Ca^2+^ excretion, because they are mostly amino acid changing and occur frequently only in one of the two groups with extreme urinary Ca^2+^ excretion. Identification of the individuals carrying these very rare variants and testing their relatives (for degree of urinary Ca^2+^ excretion and presence of variant) would help to answer if these variants contribute to the phenotype. Examining the conservation of the affected residue across species as well as the *in vitro* and *in vivo* effects on gene function would be necessary to postulate a cause-effect relation for these very rare variants.

We also analyzed our data for rare variants with allele frequency of 2–5%. Of these, the non-synonymous CLDN14 SNP rs113831133 (minor allele frequency for ∼3.3% for c.11C>T, p.Thr4Met, dbSNP) showed a lower allele frequency in individuals with high urinary Ca^2+^ excretion (∼1.1% vs. 4.1% in the lower urinary Ca^2+^ group), suggesting that if present it may lower urinary Ca^2+^ excretion, probably by decrease in CLDN14 function. The functional significance of the CLDN14 SNP rs113831133 in the thick ascending limb is unknown. Since heterozygous CLDN14-deficient mice have no renal phenotype, a dominant negative effect of rs113831133 appears more likely than haploinsufficiency [28]. The rs113831133 finding is in particular interesting since the synonymous CLDN14 SNP rs219780 has been implicated previously in kidney stone disease [27]. The frequency of this SNP was not significantly different in our study groups, though our study included a smaller sample size and our focus was on urine Ca^2+^ excretion rather than kidney stone formation. There are several different studies implicating an important role for CLDN14 in urinary Ca^2+^ excretion. CLDN14 has been shown to be a negative regulator of the CLDN16/19 complex in the TAL, which has an important role in the paracellular Ca^2+^ reabsorption in the TAL [28]. Mutations in both CLDN16 and 19 have been shown to cause familial hypercalciuria in human. In addition, recent animal studies showed a significant role for CLDN14 in urinary Ca^2+^ reabsorption [29], [30]. Homozygous CLDN14-deficient mice display lower urinary Ca^2+^ excretion than wild-type controls when challenged with a high calcium diet [28]. Renal tubule-specific Casr-deficient mice display decreased urinary Ca^2+^ excretion compared to control animals. CLDN14 is significantly downregulated (∼80%) in this mouse model [31].

Our study had several limitations. The selection of candidate genes was biased and several other candidate genes potentially related to urinary Ca^2+^ excretion were not included in our investigation. Therefore, an unbiased approach including all genes would be most favorable. This could be accomplished by exome capture followed by next-generation sequencing including all coding regions of the genome. Another limitation is the relatively low sample number reducing the power to test the main hypothesis of this study. In order to further examine the role of rare, potentially functional variants, e.g. with minor allele frequency of 1% or lower, a larger sample group is needed. Our study was designed to include sequencing data of 960 individuals, however, due to technical difficulties we were only able to include 615 samples.

## Conclusions

Our study does not support the hypothesis that rare, presumably functional allelic variants in the tested genes influence urinary Ca^2+^ excretion. Although an association of CLDN14 with urinary Ca^2+^ excretion was observed, this finding didn't reach statistical significance. Yet, our data combined with the previous GWAS, recent human and animal data suggest a potential role for CLDN14 in urinary Ca^2+^ excretion. Further studies of CLDN14 are required to study its contribution to urinary Ca^2+^ excretion and calcium-based kidney stone disease.

## Supporting Information

Table S1 **Novel SNPs identified in low and high urinary Ca^2+^ excretion cohorts.**
(DOCX)
