# Supplementary material for: Frequency of Rare Allelic Variation in Candidate Genes among Individuals with Low and High Urinary Calcium Excretion
Source: PLoS One. 2013 Aug 26;8(8):e71885. doi: 10.1371/journal.pone.0071885 (PMC3753300; doi:10.1371/journal.pone.0071885)
Supplement: Table S1 — Novel SNPs identified in low and high urinary Ca2+ excretion cohorts. (DOCX) [file pone.0071885.s001.docx]

**Table S1: Novel SNPs identified in low and high urinary Ca^2+^ excretion cohorts**

| Gene | Chr/Position | Nucleotide | Gene model | Protein | ClinVar ID | Low  UCa^2+^ | High  UCa^2+^ |
| --- | --- | --- | --- | --- | --- | --- | --- |
| ACLY | 17/40054036 | c.1395G>A | NM_001096 | p.A465A | SCV000077344 | 0.1%(1/710) | 0.0%(0/520) |
| ACLY | 17/40075176 | c.-67C>T | NM_001096 | 0 | SCV000077342 | 0.2%(1/710) | 0.0%(0/520) |
| ACLY | 17/40048537 | c.1765G>A | NM_001096 | p.A589T | SCV000077346 | 0.1%(1/710) | 0.0%(0/520) |
| ACLY | 17/40058010 | c.1122A>C | NM_001096 | p.T374T | SCV000077343 | 0.0%(0/710) | 0.2%(1/520) |
| ACLY | 17/40062858 | c.789C>T | NM_001096 | p.S263S | SCV000077351 | 0.1%(1/710) | 0.0%(0/520) |
| ACLY | 17/40065834 | c.465C>A | NM_001096 | p.G155G | SCV000077349 | 0.2%(1/710) | 0.0%(0/520) |
| ACLY | 17/40028331 | c.2747C>T | NM_001096 | p.A916V | SCV000077347 | 0.0%(0/710) | 0.2%(1/520) |
| ACLY | 17/40049403 | c.1484G>A | NM_001096 | p.R495H | SCV000077345 | 0.2%(2/710) | 0.1%(1/520) |
| ACLY | 17/40063790 | c.652G>A | NM_001096 | p.A218T | SCV000077350 | 0.0%(0/710) | 0.4%(2/520) |
| ACLY | 17/40027984 | c.2895G>A | NM_001096 | p.K965K | SCV000077348 | 0.2%(2/710) | 0.0%(0/520) |
| ACLY | 17/40025384 | c.3052-6 | NM_001096 | 0 | - | 0.2%(1/710) | 0.1%(0/520) |
| ACLY | 17/40055041 | c.1184-3 | NM_001096 | 0 | - | 0.0%(0/710) | 0.2%(1/520) |
| ACO2 | 22/41903871 | c.250C>T | NM_001098 | p.R84X | SCV000077354 | 0.2%(1/710) | 0.0%(0/520) |
| ACO2 | 22/41922431 | c.1927G>A | NM_001098 | p.V643I | SCV000077353 | 0.0%(0/710) | 0.4%(2/520) |
| ACO2 | 22/41911996 | G>A | ENST00000396512 | p.G304S | - | 0.1%(1/710) | 0.0%(0/520) |
| ACO2 | 22/41922280 | c.1776T>C | NM_001098 | p.C592C | SCV000077352 | 0.0%(0/710) | 0.2%(1/520) |
| CASR | 3/121994676 | c.1395G>A | NM_000388 | p.R465R | SCV000077306 | 0.1%(1/710) | 0.0%(0/520) |
| CASR | 3/121980951 | c.1069A>T | NM_000388 | p.N357Y | SCV000077303 | 0.1%(1/710) | 0.3%(2/520) |
| CASR | 3/121981074 | c.1192G>A | NM_000388 | p.D398N | SCV000077304 | 0.1%(1/710) | 0.0%(0/520) |
| CASR | 3/122003908 | c.3107T>C | NM_000388 | p.V1036A | SCV000077313 | 0.1%(1/710) | 0.0%(0/520) |
| CASR | 3/121980383 | c.501T>C | NM_000388 | p.Y167Y | SCV000077314 | 0.0%(0/710) | 0.2%(1/520) |
| CASR | 3/122003745 | c.2944C>T | NM_000388 | p.P982S | SCV000077312 | 0.0%(0/710) | 0.4%(2/520) |
| CASR | 3/122003721 | c.2920A>T | NM_000388 | p.T974S | SCV000077311 | 0.0%(0/710) | 0.3%(2/520) |
| CASR | 3/121980802 | c.920T>C | NM_000388 | p.M307T | SCV000077316 | 0.1%(1/710) | 0.0%(0/520) |
| CASR | 3/122003578 | c.2777A>G | NM_000388 | p.Q926R | SCV000077310 | 0.1%(1/710) | 0.0%(0/520) |
| CASR | 3/121973135 | c.99C>T | NM_000388 | p.I33I | SCV000077317 | 0.0%(0/710) | 0.2%(1/520) |
| CASR | 3/122000980 | c.1629C>T | NM_000388 | p.S543S | SCV000077307 | 0.0%(0/710) | 0.4%(2/520) |
| CASR | 3/121980622 | c.740C>T | NM_000388 | p.S247F | SCV000077315 | 0.1%(1/710) | 0.0%(0/520) |
| CASR | 3/121981091 | c.1209T>C | NM_000388 | p.S403S | SCV000077305 | 0.2%(1/710) | 0.1%(1/520) |
| CASR | 3/122003398 | c.2597G>T | NM_000388 | p.R866L | SCV000077309 | 0.1%(1/710) | 0.2%(1/520) |
| CASR | 3/122001019 | c.1668G>A | NM_000388 | p.E556E | SCV000077308 | 0.0%(0/710) | 0.2%(1/520) |
| CLCN5 | X/49846438 | c.657C>T | NM_000084 | p.C219C | SCV000077252 | 0.2%(1/710) | 0.0%(0/520) |
| CLCN5 | X/49854856 | c.1618G>A | NM_000084 | p.A540T | SCV000077250 | 0.1%(1/710) | 0.0%(0/520) |
| CLCN5 | X/49855012 | c.1774G>A | NM_000084 | p.D592N | SCV000077251 | 0.1%(1/710) | 0.0%(0/520) |
| CLCNKA | 1/16355714 | c.1147G>T | NM_001042704 | p.D383Y | SCV000077336 | 0.2%(1/710) | 0.0%(0/520) |
| CLCNKA | 1/16353902 | c.753G>C | NM_001042704 | p.R251R | SCV000077340 | 0.0%(0/710) | 0.2%(1/520) |
| CLCNKA | 1/16353903 | c.754C>T | NM_001042704 | p.L252F | SCV000077341 | 0.3%(2/710) | 0.0%(0/520) |
| CLCNKA | 1/16359712 | c.1974A>G | NM_001042704 | p.T658T | SCV000077338 | 0.2%(1/710) | 0.0%(0/520) |
| CLCNKA | 1/16356489 | c.1327G>A | NM_001042704 | p.A443T | SCV000077337 | 0.1%(1/710) | 0.2%(1/520) |
| CLCNKA | 1/16349188 | c.74G>A | NM_001042704 | p.C25Y | SCV000077339 | 0.0%(0/710) | 0.2%(1/520) |
| CLCNKB | 1/16380186 | c.1675G>A | NM_000085 | p.A559T | SCV000077254 | 0.2%(1/710) | 0.0%(0/520) |
| CLCNKB | 1/16382163 | c.1846-7 | NM_000085 | 0 | - | 0.1%(1/710) | 0.3%(2/520) |
| CLCNKB | 1/16375516 | A>G | ENST00000375667 | pQ17R | - | 0.0%(0/710) | 0.0%(0/520) |
| CLCNKB | 1/16371010 | c.23G>A | NM_000085 | p.R8H | SCV000077257 | 0.0%(0/710) | 0.2%(1/520) |
| CLCNKB | 1/16375471 | C>T | ENST00000375667 | p.P2L | - | 0.3%(2/710) | 0.1%(0/520) |
| CLCNKB | 1/16378219 | c.1312C>T | NM_000085 | p.R438C | SCV000077253 | 0.3%(2/710) | 0.0%(0/520) |
| CLCNKB | 1/16382931 | c.1944T>G | NM_000085 | p.F648L | SCV000077255 | 0.3%(2/710) | 0.0%(0/520) |
| CLCNKB | 1/16382989 | c.2002G>C | NM_000085 | p.V668L | SCV000077256 | 0.1%(1/710) | 0.0%(0/520) |
| CLCNKB | 1/16375564 | T>C | ENST00000375667 | p.V33A | - | 0.0%(0/710) | 0.2%(1/520) |
| CLCNKB | 1/16371058 | c.71C>T | NM_000085 | p.P24L | SCV000077258 | 0.1%(1/710) | 0.0%(0/520) |
| CLDN10 | 13/96212673 | c.357C>A | NM_001160100 | p.N119K | SCV000077368 | 0.1%(1/710) | 0.0%(0/520) |
| CLDN10 | 13/96212724 | c.401+7 | NM_001160100 | 0 | - | 0.2%(1/710) | 0.0%(0/520) |
| CLDN10 | 13/96229525 | c.442G>T | NM_001160100 | p.A148S | SCV000077369 | 0.0%(0/710) | 0.2%(1/520) |
| CLDN10 | 13/96205123 | T>C | ENST00000299339 | p.V39A | - | 0.0%(0/710) | 0.2%(1/520) |
| CLDN10 | 13/96212483 | c.255C>T | NM_001160100 | p.V85V | SCV000077367 | 0.1%(1/710) | 0.0%(0/520) |
| CLDN14 | 21/37833331 | c.663G>A | NM_001146077 | p.R221R | SCV000077366 | 0.0%(0/710) | 0.2%(1/520) |
| CLDN14 | 21/37833934 | c.60C>T | NM_001146077 | p.G20G | SCV000077365 | 0.1%(1/710) | 0.2%(1/520) |
| CLDN14 | 21/37833979 | c.15C>T | NM_001146077 | p.A5A | SCV000077363 | 0.0%(0/710) | 0.2%(1/520) |
| CLDN14 | 21/37833976 | c.18G>A | NM_001146077 | p.V6V | SCV000077364 | 0.3%(2/710) | 0.0%(0/520) |
| CLDN19 | 1/43205582 | c.153G>T | NM_001123395 | p.W51C | SCV000077357 | 0.0%(0/710) | 0.2%(1/520) |
| CLDN19 | 1/43203959 | C>T | ENST00000296387 | p.S138 | - | 0.2%(1/710) | 0.0%(0/520) |
| CLDN19 | 1/43204179 | c.301G>A | NM_001123395 | p.G101S | SCV000077358 | 0.1%(1/710) | 0.0%(0/520) |
| CLDN19 | 1/43200761 | c.*778T>A | NM_001123395 | 0 | SCV000077355 | 0.0%(0/710) | 0.2%(1/520) |
| CLDN19 | 1/43205607 | c.128C>T | NM_001123395 | p.A43V | SCV000077356 | 0.0%(0/710) | 0.2%(1/520) |
| CLDN2 | X/106171980 | c.522C>T | NM_001171092 | p.S174S | SCV000077373 | 0.3%(2/710) | 0.0%(0/520) |
| CLDN2 | X/106171651 | c.193G>C | NM_001171092 | p.D65H | SCV000077372 | 0.3%(2/710) | 0.0%(0/520) |
| CLDN8 | 21/31588082 | c.162C>A | NM_199328 | p.C54X | SCV000077482 | 0.0%(0/710) | 0.2%(1/520) |
| CLDN8 | 21/31587886 | c.358C>T | NM_199328 | p.L120L | SCV000077483 | 0.3%(2/710) | 0.0%(0/520) |
| FGF23 | 12/4479642 | c.623A>G | NM_020638 | p.Q208R | SCV000077452 | 0.1%(1/710) | 0.0%(0/520) |
| GCM2 | 6/10874916 | c.833C>T | NM_004752 | p.A278V | SCV000077419 | 0.1%(1/710) | 0.0%(0/520) |
| GCM2 | 6/10874918 | c.831G>T | NM_004752 | p.L277F | SCV000077418 | 0.1%(1/710) | 0.0%(0/520) |
| GCM2 | 6/10874843 | c.906A>T | NM_004752 | p.T302T | SCV000077421 | 0.1%(1/710) | 0.4%(2/520) |
| GCM2 | 6/10874897 | c.852T>C | NM_004752 | p.N284N | SCV000077420 | 0.0%(0/710) | 0.2%(1/520) |
| GCM2 | 6/10875084 | c.665A>T | NM_004752 | p.E222V | SCV000077416 | 0.1%(1/710) | 0.4%(2/520) |
| GCM2 | 6/10874967 | c.782A>T | NM_004752 | p.Y261F | SCV000077417 | 0.3%(2/710) | 0.1%(1/520) |
| GCM2 | 6/10876229 | c.477T>C | NM_004752 | p.H159H | SCV000077415 | 0.1%(1/710) | 0.0%(0/520) |
| GCM2 | 6/10877413 | c.303G>T | NM_004752 | p.L101L | SCV000077414 | 0.1%(1/710) | 0.0%(0/520) |
| GCM2 | 6/10874472 | c.1277A>C | NM_004752 | p.Y426S | SCV000077413 | 0.1%(1/710) | 0.3%(1/520) |
| GCM2 | 6/10874581 | c.1168T>C | NM_004752 | p.S390P | SCV000077412 | 0.0%(0/710) | 0.2%(1/520) |
| KCNJ1 | 11/128709111 | c.1085A>C | NM_000220 | p.E362A | SCV000077266 | 0.0%(0/710) | 0.4%(2/520) |
| KCNJ1 | 11/128709112 | c.1084G>A | NM_000220 | p.E362K | SCV000077265 | 0.1%(1/710) | 0.2%(1/520) |
| KCNJ1 | 11/128709015 | c.*5G>A | NM_000220 | 0 | SCV000077264 | 0.1%(1/710) | 0.0%(0/520) |
| KCNJ1 | 11/128710041 | c.155A>C | NM_000220 | p.E52A | SCV000077267 | 0.2%(1/710) | 0.1%(0/520) |
| KCNJ1 | 11/128709877 | c.319A>G | NM_000220 | p.K107E | SCV000077269 | 0.0%(0/710) | 0.2%(1/520) |
| KCNJ1 | 11/128709897 | c.299A>G | NM_000220 | p.Y100C | SCV000077268 | 0.0%(0/710) | 0.2%(1/520) |
| KCNJ1 | 11/128709420 | c.776A>C | NM_000220 | p.N259T | SCV000077270 | 0.0%(0/710) | 0.3%(2/520) |
| KL | 13/33635900 | c.2684T>C | NM_004795 | p.I895T | SCV000077424 | 0.1%(1/710) | 0.0%(0/520) |
| KL | 13/33635721 | c.2505C>G | NM_004795 | p.D835E | SCV000077422 | 0.2%(2/710) | 0.4%(2/520) |
| KL | 13/33635899 | c.2683A>G | NM_004795 | p.I895V | SCV000077423 | 0.3%(2/710) | 0.0%(0/520) |
| KL | 13/33591075 | c.497A>G | NM_004795 | p.N166S | SCV000077428 | 0.2%(1/710) | 0.0%(0/520) |
| KL | 13/33591217 | c.639C>A | NM_004795 | p.A213A | SCV000077429 | 0.0%(0/710) | 0.2%(1/520) |
| KL | 13/33638313 | c.3029G>A | NM_004795 | p.S1010N | SCV000077426 | 0.1%(1/710) | 0.0%(0/520) |
| KL | 13/33638146 | c.2862G>A | NM_004795 | p.P954P | SCV000077425 | 0.1%(1/710) | 0.0%(0/520) |
| KL | 13/33590965 | c.387C>T | NM_004795 | p.Y129Y | SCV000077427 | 0.1%(1/710) | 0.2%(1/520) |
| PDZK1 | 1/145747105 | c.62G>A | NM_002614 | p.G21D | SCV000077383 | 0.1%(1/710) | 0.0%(0/520) |
| PIK3C2G | 12/18466938 | c.1077A>T | NM_004570 | p.K359N | SCV000077405 | 0.0%(0/710) | 0.2%(1/520) |
| PIK3C2G | 12/18644413 | c.2591T>G | NM_004570 | p.L864R | SCV000077406 | 0.0%(0/710) | 0.4%(2/520) |
| PIK3C2G | 12/18691225 | c.3336G>A | NM_004570 | p.L1112L | SCV000077409 | 0.2%(1/710) | 0.0%(0/520) |
| PIK3C2G | 12/18716346 | c.3693A>G | NM_004570 | p.E1231E | SCV000077410 | 0.3%(2/710) | 0.0%(0/520) |
| PIK3C2G | 12/18716352 | c.3699C>A | NM_004570 | p.S1233R | SCV000077411 | 0.1%(1/710) | 0.0%(0/520) |
| PIK3C2G | 12/18649011 | c.2686A>T | NM_004570 | p.T896S | SCV000077407 | 0.2%(1/710) | 0.0%(0/520) |
| PIK3C2G | 12/18650584 | c.2795T>C | NM_004570 | p.V932A | SCV000077408 | 0.0%(0/710) | 0.2%(1/520) |
| PTH | 11/13514178 | c.122A>G | NM_000315 | p.N41S | SCV000077271 | 0.2%(1/710) | 0.0%(0/520) |
| PTH | 11/13514000 | c.300G>T | NM_000315 | p.E100D | SCV000077272 | 0.0%(0/710) | 0.1%(1/520) |
| PTH1R | 3/46940191 | c.678G>T | NM_000316 | p.L226L | SCV000077278 | 0.2%(2/710) | 0.4%(2/520) |
| PTH1R | 3/46939665 | c.526A>T | NM_000316 | p.N176Y | SCV000077277 | 0.3%(2/710) | 0.0%(0/520) |
| PTH1R | 3/46935445 | c.124C>T | NM_000316 | p.H42Y | SCV000077274 | 0.2%(1/710) | 0.0%(0/520) |
| PTH1R | 3/46943294 | c.1155C>T | NM_000316 | p.L385L | SCV000077273 | 0.0%(0/710) | 0.3%(2/520) |
| PTH1R | 3/46940833 | c.875T>C | NM_000316 | p.L292P | SCV000077280 | 0.2%(1/710) | 0.0%(0/520) |
| PTH1R | 3/46944108 | c.1304C>T | NM_000316 | p.T435M | SCV000077275 | 0.2%(1/710) | 0.0%(0/520) |
| PTH1R | 3/46939347 | c.316C>T | NM_000316 | p.R106C | SCV000077276 | 0.2%(1/710) | 0.2%(1/520) |
| PTH1R | 3/46935392 | c.76-5 | NM_000316 | 0 | - | 0.4%(3/710) | 0.1%(1/520) |
| PTH1R | 3/46940318 | c.805C>T | NM_000316 | p.P269S | SCV000077279 | 0.2%(1/710) | 0.0%(0/520) |
| SLC12A1 | 15/48559779 | c.2176A>T | NM_000338 | p.K726X | SCV000077284 | 0.2%(2/710) | 0.0%(0/520) |
| SLC12A1 | 15/48559780 | c.2177A>T | NM_000338 | p.K726M | SCV000077285 | 0.3%(2/710) | 0.0%(0/520) |
| SLC12A1 | 15/48512920 | c.510T>A | NM_000338 | p.D170E | SCV000077289 | 0.2%(1/710) | 0.2%(1/520) |
| SLC12A1 | 15/48499929 | c.13A>G | NM_000338 | p.N5D | SCV000077282 | 0.1%(1/710) | 0.4%(2/520) |
| SLC12A1 | 15/48512913 | c.503A>G | NM_000338 | p.E168G | SCV000077288 | 0.1%(1/710) | 0.0%(0/520) |
| SLC12A1 | 15/48559865 | c.2262C>T | NM_000338 | p.D754D | SCV000077286 | 0.2%(1/710) | 0.0%(0/520) |
| SLC12A1 | 15/48500302 | c.386C>T | NM_000338 | p.P129L | SCV000077287 | 0.0%(0/710) | 0.2%(1/520) |
| SLC12A1 | 15/48527135 | c.1149C>T | NM_000338 | p.V383V | SCV000077281 | 0.1%(1/710) | 0.0%(0/520) |
| SLC12A1 | 15/48548063 | c.1998T>C | NM_000338 | p.N666N | SCV000077283 | 0.1%(1/710) | 0.2%(1/520) |
| SLC12A1 | 15/48527205 | c.1215+4 | NM_000338 | 0 | - | 0.1%(1/710) | 0.0%(0/520) |
| SLC12A3 | 16/56904103 | c.697A>G | NM_000339 | p.M233V | SCV000077295 | 0.2%(2/710) | 0.0%(0/520) |
| SLC12A3 | 16/56913547 | c.1429G>A | NM_000339 | p.A477T | SCV000077291 | 0.0%(0/710) | 0.2%(1/520) |
| SLC12A3 | 16/56924210 | c.2310C>T | NM_000339 | p.G770G | SCV000077294 | 0.1%(1/710) | 0.4%(2/520) |
| SLC12A3 | 16/56918001 | c.1710G>A | NM_000339 | p.A570A | SCV000077292 | 0.0%(0/710) | 0.3%(1/520) |
| SLC12A3 | 16/56920306 | c.1956C>A | NM_000339 | p.P652P | SCV000077293 | 0.1%(1/710) | 0.2%(1/520) |
| SLC12A3 | 16/56912993 | c.1189G>A | NM_000339 | p.V397M | SCV000077290 | 0.0%(0/710) | 0.4%(2/520) |
| SLC12A3 | 16/56926860 | c.2447-5 | NM_000339 | 0 | - | 0.2%(1/710) | 0.0%(0/520) |
| SLC12A3 | 16/56906271 | c.861G>T | NM_000339 | p.V287V | SCV000077296 | 0.1%(1/710) | 0.4%(2/520) |
| SLC13A2 | 17/26822734 | c.1517C>T | NM_001145975 | p.P506L | SCV000077362 | 0.1%(1/710) | 0.0%(0/520) |
| SLC13A2 | 17/26822690 | c.1473G>A | NM_001145975 | p.E491E | SCV000077361 | 0.1%(1/710) | 0.0%(0/520) |
| SLC13A2 | 17/26821081 | T>C | ENST00000537681 | p.F311L | - | 0.0%(0/710) | 0.1%(1/520) |
| SLC13A3 | 20/45242184 | c.151A>T | NM_001011554 | p.I51F | SCV000077325 | 0.1%(1/710) | 0.0%(0/520) |
| SLC25A1 | 22/19164141 | c.697G>A | NM_005984 | p.A233T | SCV000077440 | 0.1%(1/710) | 0.0%(0/520) |
| SLC25A1 | 22/19165471 | c.286C>T | NM_005984 | p.P96S | SCV000077438 | 0.1%(1/710) | 0.0%(0/520) |
| SLC25A1 | 22/19165710 | c.138C>A | NM_005984 | p.T46T | SCV000077437 | 0.3%(2/710) | 0.0%(0/520) |
| SLC25A1 | 22/19164375 | c.615G>A | NM_005984 | p.L205L | SCV000077439 | 0.1%(1/710) | 0.4%(2/520) |
| SLC26A1 | 4/983218 | c.1509C>T | NM_022042 | p.G503G | SCV000077459 | 0.1%(1/710) | 0.0%(0/520) |
| SLC26A1 | 4/983321 | c.1406C>T | NM_022042 | p.A469V | SCV000077456 | 0.0%(0/710) | 0.2%(1/520) |
| SLC26A1 | 4/983016 | c.1711G>A | NM_022042 | p.A571T | SCV000077463 | 0.0%(0/710) | 0.2%(1/520) |
| SLC26A1 | 4/985266 | c.226G>A | NM_022042 | p.G76R | SCV000077466 | 0.1%(1/710) | 0.0%(0/520) |
| SLC26A1 | 4/981637 | c.*984T>A | NM_022042 | 0 | SCV000077453 | 0.1%(1/710) | 0.0%(0/520) |
| SLC26A1 | 4/983364 | c.1363C>T | NM_022042 | p.R455C | SCV000077455 | 0.0%(0/710) | 0.4%(2/520) |
| SLC26A1 | 4/983105 | c.1622G>A | NM_022042 | p.R541H | SCV000077460 | 0.0%(0/710) | 0.3%(1/520) |
| SLC26A1 | 4/983967 | c.760C>T | NM_022042 | p.R254C | SCV000077470 | 0.3%(2/710) | 0.0%(0/520) |
| SLC26A1 | 4/982841 | c.1886C>T | NM_022042 | p.T629M | SCV000077464 | 0.2%(1/710) | 0.0%(0/520) |
| SLC26A1 | 4/983094 | c.1633C>T | NM_022042 | p.P545S | SCV000077461 | 0.0%(0/710) | 0.2%(1/520) |
| SLC26A1 | 4/985059 | c.433G>A | NM_022042 | p.G145S | SCV000077468 | 0.0%(0/710) | 0.3%(2/520) |
| SLC26A1 | 4/982821 | c.1906G>T | NM_022042 | p.D636Y | SCV000077465 | 0.3%(2/710) | 0.0%(0/520) |
| SLC26A1 | 4/983279 | c.1448T>A | NM_022042 | p.L483Q | SCV000077457 | 0.1%(1/710) | 0.0%(0/520) |
| SLC26A1 | 4/985136 | c.356G>A | NM_022042 | p.R119Q | SCV000077467 | 0.2%(1/710) | 0.0%(0/520) |
| SLC26A1 | 4/983268 | c.1459G>A | NM_022042 | p.E487K | SCV000077458 | 0.2%(1/710) | 0.2%(1/520) |
| SLC26A1 | 4/983042 | c.1685C>T | NM_022042 | p.T562M | SCV000077462 | 0.2%(1/710) | 0.2%(1/520) |
| SLC26A1 | 4/983996 | c.731T>C | NM_022042 | p.M244T | SCV000077469 | 0.1%(1/710) | 0.0%(0/520) |
| SLC26A1 | 4/983684 | c.1043T>C | NM_022042 | p.L348P | SCV000077454 | 0.0%(0/710) | 0.4%(2/520) |
| SLC26A2 | 5/149360371 | c.1215C>G | NM_000112 | p.A405A | SCV000077259 | 0.1%(1/710) | 0.4%(2/520) |
| SLC26A2 | 5/149361152 | c.1996A>G | NM_000112 | p.T666A | SCV000077262 | 0.0%(0/710) | 0.2%(1/520) |
| SLC26A2 | 5/149357296 | c.81C>G | NM_000112 | p.I27M | SCV000077263 | 0.2%(1/710) | 0.2%(1/520) |
| SLC26A2 | 5/149357387 | c.172C>T | NM_000112 | p.R58C | SCV000077260 | 0.1%(1/710) | 0.0%(0/520) |
| SLC26A2 | 5/149360954 | c.1798T>C | NM_000112 | p.Y600H | SCV000077261 | 0.3%(2/710) | 0.1%(1/520) |
| SLC26A6 | 3/48664480 | c.1839C>T | NM_001040454 | p.S613S | SCV000077331 | 0.2%(1/710) | 0.2%(1/520) |
| SLC26A6 | 3/48664492 | c.1831-4 | NM_001040454 | 0 | - | 0.0%(0/710) | 0.2%(1/520) |
| SLC26A6 | 3/48666129 | c.1555G>C | NM_001040454 | p.V519L | SCV000077328 | 0.1%(1/710) | 0.0%(0/520) |
| SLC26A6 | 3/48666116 | c.1568G>A | NM_001040454 | p.R523H | SCV000077329 | 0.0%(0/710) | 0.2%(1/520) |
| SLC26A6 | 3/48669354 | c.646C>A | NM_001040454 | p.L216I | SCV000077335 | 0.3%(2/710) | 0.2%(1/520) |
| SLC26A6 | 3/48670475 | c.302G>A | NM_001040454 | p.G101D | SCV000077333 | 0.0%(0/710) | 0.2%(1/520) |
| SLC26A6 | 3/48669381 | c.619G>C | NM_001040454 | p.V207L | SCV000077334 | 0.0%(0/710) | 0.2%(1/520) |
| SLC26A6 | 3/48665388 | G>A | ENST00000337000 | p.C521 | - | 0.1%(1/710) | 0.0%(0/520) |
| SLC26A6 | 3/48667133 | c.1478C>T | NM_001040454 | p.S493F | SCV000077326 | 0.0%(0/710) | 0.2%(1/520) |
| SLC26A6 | 3/48664111 | c.2018A>T | NM_001040454 | p.H673L | SCV000077332 | 0.1%(1/710) | 0.5%(3/520) |
| SLC26A6 | 3/48667108 | c.1503G>A | NM_001040454 | p.T501T | SCV000077327 | 0.0%(0/710) | 0.2%(1/520) |
| SLC26A6 | 3/48666094 | c.1590T>A | NM_001040454 | p.F530L | SCV000077330 | 0.0%(0/710) | 0.2%(1/520) |
| SLC34A1 | 5/176825279 | c.1912C>T | NM_003052 | p.R638C | SCV000077400 | 0.3%(2/710) | 0.0%(0/520) |
| SLC34A1 | 5/176825106 | c.1739C>A | NM_003052 | p.P580H | SCV000077399 | 0.2%(1/710) | 0.3%(1/520) |
| SLC34A1 | 5/176813352 | c.388+2 | NM_001167579 | 0 | - | 0.0%(0/710) | 0.2%(1/520) |
| SLC34A1 | 5/176815010 | c.660C>G | NM_001167579 | p.A220A | SCV000077371 | 0.1%(1/710) | 0.0%(0/520) |
| SLC34A1 | 5/176824799 | c.1432T>C | NM_003052 | p.F478L | SCV000077397 | 0.3%(2/710) | 0.2%(1/520) |
| SLC34A1 | 5/176813265 | c.303G>A | NM_001167579 | p.L101L | SCV000077370 | 0.1%(1/710) | 0.0%(0/520) |
| SLC34A1 | 5/176814875 | c.644+1 | NM_001167579 | 0 | - | 0.0%(0/710) | 0.4%(2/520) |
| SLC34A1 | 5/176825074 | c.1707G>T | NM_003052 | p.L569L | SCV000077398 | 0.0%(0/710) | 0.2%(1/520) |
| SLC34A3 | 9/140128917 | c.1143G>A | NM_001177316 | p.A381A | SCV000077375 | 0.1%(1/710) | 0.0%(0/520) |
| SLC34A3 | 9/140127672 | c.572G>A | NM_001177316 | p.G191D | SCV000077381 | 0.2%(1/710) | 0.0%(0/520) |
| SLC34A3 | 9/140127096 | c.245G>T | NM_001177316 | p.S82I | SCV000077377 | 0.2%(2/710) | 0.2%(1/520) |
| SLC34A3 | 9/140127157 | c.304+2 | NM_001177316 | 0 | - | 0.1%(1/710) | 0.4%(2/520) |
| SLC34A3 | 9/140127479 | c.472C>T | NM_001177316 | p.P158S | SCV000077379 | 0.1%(1/710) | 0.0%(0/520) |
| SLC34A3 | 9/140127069 | c.218T>C | NM_001177316 | p.V73A | SCV000077376 | 0.1%(1/710) | 0.0%(0/520) |
| SLC34A3 | 9/140127370 | c.439G>A | NM_001177316 | p.A147T | SCV000077378 | 0.0%(0/710) | 0.2%(1/520) |
| SLC34A3 | 9/140127523 | c.516C>G | NM_001177316 | p.T172T | SCV000077380 | 0.0%(0/710) | 0.2%(1/520) |
| SLC34A3 | 9/140128686 | c.1051C>T | NM_001177316 | p.R351C | SCV000077374 | 0.0%(0/710) | 0.3%(1/520) |
| SLC34A3 | 9/140127685 | c.585C>T | NM_001177316 | p.H195H | SCV000077382 | 0.2%(1/710) | 0.3%(1/520) |
| SLC4A1 | 17/42335174 | c.1284A>G | NM_000342 | p.G428G | SCV000077297 | 0.1%(1/710) | 0.0%(0/520) |
| SLC4A1 | 17/42334919 | c.1432-7 | NM_000342 | 0 | - | 0.0%(0/710) | 0.2%(1/520) |
| SLC4A1 | 17/42335083 | c.1375C>T | NM_000342 | p.L459L | SCV000077298 | 0.2%(1/710) | 0.0%(0/520) |
| SLC4A1 | 17/42327868 | c.2694G>A | NM_000342 | p.E898E | SCV000077299 | 0.1%(1/710) | 0.0%(0/520) |
| SLC4A1 | 17/42327861 | c.2701C>T | NM_000342 | p.R901W | SCV000077300 | 0.1%(1/710) | 0.0%(0/520) |
| SLC4A2 | 7/150773178 | c.3550G>A | NM_003040 | p.A1184T | SCV000077391 | 0.1%(1/710) | 0.0%(0/520) |
| SLC4A2 | 7/150768826 | c.2242G>T | NM_003040 | p.A748S | SCV000077385 | 0.2%(1/710) | 0.0%(0/520) |
| SLC4A2 | 7/150772733 | c.3342C>T | NM_003040 | p.P1114P | SCV000077388 | 0.2%(1/710) | 0.0%(0/520) |
| SLC4A2 | 7/150772434 | c.3140C>T | NM_003040 | p.A1047V | SCV000077387 | 0.3%(2/710) | 0.0%(0/520) |
| SLC4A2 | 7/150761331 | c.94G>A | NM_003040 | p.E32K | SCV000077396 | 0.0%(0/710) | 0.2%(1/520) |
| SLC4A2 | 7/150773102 | c.3474C>T | NM_003040 | p.V1158V | SCV000077390 | 0.1%(1/710) | 0.0%(0/520) |
| SLC4A2 | 7/150761632 | c.237C>T | NM_003040 | p.H79H | SCV000077386 | 0.1%(1/710) | 0.0%(0/520) |
| SLC4A2 | 7/150761290 | c.53C>T | NM_003040 | p.P18L | SCV000077393 | 0.2%(1/710) | 0.2%(1/520) |
| SLC4A2 | 7/150761298 | c.61G>A | NM_003040 | p.E21K | SCV000077394 | 0.2%(1/710) | 0.0%(0/520) |
| SLC4A2 | 7/150772749 | c.3358G>A | NM_003040 | p.G1120R | SCV000077389 | 0.3%(2/710) | 0.0%(0/520) |
| SLC4A2 | 7/150763671 | c.646G>A | NM_003040 | p.G216R | SCV000077395 | 0.0%(0/710) | 0.2%(1/520) |
| SLC4A2 | 7/150773187 | c.3559C>T | NM_003040 | p.L1187L | SCV000077392 | 0.0%(0/710) | 0.2%(1/520) |
| SLC4A2 | 7/150768804 | c.2220G>T | NM_003040 | p.V740V | SCV000077384 | 0.1%(1/710) | 0.0%(0/520) |
| SLC4A3 | 2/220497995 | c.1278-1 | NM_005070 | 0 | - | 0.0%(0/710) | 0.2%(1/520) |
| SLC4A3 | 2/220504246 | c.3066C>T | NM_005070 | p.S1022S | SCV000077433 | 0.0%(0/710) | 0.2%(1/520) |
| SLC4A3 | 2/220493221 | c.146A>G | NM_005070 | p.D49G | SCV000077430 | 0.0%(0/710) | 0.2%(1/520) |
| SLC4A3 | 2/220494927 | c.664C>A | NM_005070 | p.P222T | SCV000077435 | 0.1%(1/710) | 0.0%(0/520) |
| SLC4A3 | 2/220494837 | G>A | ENST00000273063 | p.A219T | - | 0.0%(0/710) | 0.2%(1/520) |
| SLC4A3 | 2/220494019 | c.371C>T | NM_005070 | p.P124L | SCV000077434 | 0.0%(0/710) | 0.3%(1/520) |
| SLC4A3 | 2/220494944 | c.681C>T | NM_005070 | p.A227A | SCV000077436 | 0.0%(0/710) | 0.2%(1/520) |
| SLC4A3 | 2/220501065 | c.2233G>A | NM_005070 | p.V745M | SCV000077431 | 0.1%(1/710) | 0.0%(0/520) |
| SLC4A3 | 2/220502903 | c.2784G>A | NM_005070 | p.G928G | SCV000077432 | 0.0%(0/710) | 0.2%(1/520) |
| SLC9A3R1 | 17/72764748 | c.1030C>G | NM_004252 | p.P344A | SCV000077401 | 0.0%(0/710) | 0.2%(1/520) |
| SLC9A3R1 | 17/72759610 | c.708C>G | NM_004252 | p.D236E | SCV000077403 | 0.0%(0/710) | 0.2%(1/520) |
| SLC9A3R1 | 17/72764676 | c.958A>T | NM_004252 | p.I320F | SCV000077404 | 0.1%(1/710) | 0.3%(2/520) |
| SLC9A3R1 | 17/72745018 | c.33G>T | NM_004252 | p.L11L | SCV000077402 | 0.2%(1/710) | 0.0%(0/520) |
| SLC9A3R2 | 16/2079621 | c.252G>A | NM_001130012 | p.R84R | SCV000077359 | 0.1%(1/710) | 0.0%(0/520) |
| SLC9A3R2 | 16/2086885 | c.748+3 | NM_001130012 | 0 | - | 0.1%(1/710) | 0.0%(0/520) |
| SLC9A3R2 | 16/2086396 | c.486G>A | NM_001130012 | p.G162G | SCV000077360 | 0.2%(1/710) | 0.2%(1/520) |
| TRPV5 | 7/142605902 | c.1968G>A | NM_019841 | p.K656K | SCV000077447 | 0.0%(0/710) | 0.2%(1/520) |
| TRPV5 | 7/142626168 | c.535G>A | NM_019841 | p.V179M | SCV000077450 | 0.0%(0/710) | 0.2%(1/520) |
| TRPV5 | 7/142622780 | c.966C>T | NM_019841 | p.N322N | SCV000077451 | 0.0%(0/710) | 0.2%(1/520) |
| TRPV5 | 7/142606722 | c.1829G>A | NM_019841 | p.R610H | SCV000077446 | 0.0%(0/710) | 0.4%(2/520) |
| TRPV5 | 7/142626540 | c.470G>A | NM_019841 | p.R157H | SCV000077449 | 0.1%(1/710) | 0.0%(0/520) |
| TRPV5 | 7/142630524 | c.33C>T | NM_019841 | p.P11P | SCV000077448 | 0.4%(3/710) | 0.0%(0/520) |
| TRPV5 | 7/142622711 | c.1035G>A | NM_019841 | p.T345T | SCV000077445 | 0.2%(1/710) | 0.0%(0/520) |
| TRPV6 | 7/142575471 | c.282C>T | NM_018646 | p.A94A | SCV000077444 | 0.0%(0/710) | 0.2%(1/520) |
| TRPV6 | 7/142574339 | c.587-3 | NM_018646 | 0 | - | 0.1%(1/710) | 0.0%(0/520) |
| TRPV6 | 7/142572258 | c.1438A>C | NM_018646 | p.I480L | SCV000077442 | 0.2%(1/710) | 0.3%(2/520) |
| TRPV6 | 7/142572353 | c.1343G>A | NM_018646 | p.S448N | SCV000077441 | 0.1%(1/710) | 0.0%(0/520) |
| TRPV6 | 7/142575402 | c.349+2 | NM_018646 | 0 | - | 0.0%(0/710) | 0.3%(1/520) |
| TRPV6 | 7/142571205 | c.1784C>A | NM_018646 | p.A595D | SCV000077443 | 0.0%(0/710) | 0.2%(1/520) |
| UMOD | 16/20348027 | c.1763G>A | NM_001008389 | p.R588Q | SCV000077321 | 0.0%(0/710) | 0.2%(1/520) |
| UMOD | 16/20357602 | c.1028C>T | NM_001008389 | p.S343L | SCV000077319 | 0.0%(0/710) | 0.2%(1/520) |
| UMOD | 16/20359826 | c.797C>T | NM_001008389 | p.A266V | SCV000077323 | 0.1%(1/710) | 0.0%(0/520) |
| UMOD | 16/20359585 | c.933C>T | NM_001008389 | p.G311G | SCV000077324 | 0.1%(1/710) | 0.1%(1/520) |
| UMOD | 16/20348705 | c.1648G>A | NM_001008389 | p.V550I | SCV000077320 | 0.2%(1/710) | 0.1%(0/520) |
| UMOD | 16/20360056 | c.567C>T | NM_001008389 | p.Y189Y | SCV000077322 | 0.0%(0/710) | 0.2%(1/520) |
| UMOD | 16/20344628 | c.*8C>T | NM_001008389 | 0 | SCV000077318 | 0.2%(1/710) | 0.0%(0/520) |
| VDR | 12/48258869 | c.238C>A | NM_000376 | p.R80R | SCV000077301 | 0.0%(0/710) | 0.2%(1/520) |
| VDR | 12/48258848 | c.259A>G | NM_000376 | p.I87V | SCV000077302 | 0.2%(1/710) | 0.0%(0/520) |
| WNK4 | 17/40937468 | c.1444C>T | NM_032387 | p.R482W | SCV000077473 | 0.1%(1/710) | 0.4%(2/520) |
| WNK4 | 17/40940176 | c.1892G>A | NM_032387 | p.G631D | SCV000077474 | 0.2%(1/710) | 0.0%(0/520) |
| WNK4 | 17/40947016 | c.2577C>T | NM_032387 | p.T859T | SCV000077477 | 0.2%(1/710) | 0.0%(0/520) |
| WNK4 | 17/40936136 | c.973G>A | NM_032387 | p.A325T | SCV000077481 | 0.1%(1/710) | 0.0%(0/520) |
| WNK4 | 17/40948245 | c.3536C>T | NM_032387 | p.A1179V | SCV000077479 | 0.1%(1/710) | 0.0%(0/520) |
| WNK4 | 17/40940742 | c.2084C>G | NM_032387 | p.T695S | SCV000077475 | 0.1%(1/710) | 0.0%(0/520) |
| WNK4 | 17/40946915 | c.2476G>A | NM_032387 | p.G826S | SCV000077476 | 0.1%(1/710) | 0.0%(0/520) |
| WNK4 | 17/40937347 | c.1323A>T | NM_032387 | p.E441D | SCV000077472 | 0.1%(1/710) | 0.0%(0/520) |
| WNK4 | 17/40933262 | c.546T>A | NM_032387 | p.R182R | SCV000077480 | 0.1%(1/710) | 0.2%(1/520) |
| WNK4 | 17/40947157 | c.2718T>C | NM_032387 | p.F906F | SCV000077478 | 0.2%(1/710) | 0.0%(0/520) |
| WNK4 | 17/40937166 | c.1222A>T | NM_032387 | p.I408F | SCV000077471 | 0.0%(0/710) | 0.2%(1/520) |
